# Thyrotoxic crisis presenting with jaundice

**DOI:** 10.1186/s13104-016-2126-z

**Published:** 2016-06-23

**Authors:** R. D. S. S. Wickramasinghe, W. A. N. V. Luke, B. S. Sebastiampillai, M. P. M. L. Gunathilake, R. Premaratna

**Affiliations:** Professorial Medical Unit, Colombo North Teaching Hospital, Ragama, Sri Lanka; Department of Clinical Pharmacology, Faculty of Medicine, University of Kelaniya, Ragama, Sri Lanka; Department of Medicine, Faculty of Medicine, University of Kelaniya, Ragama, Sri Lanka

**Keywords:** Thyrotoxic crisis, Jaundice, Graves disease

## Abstract

**Background:**

Thyrotoxic crisis is a medical emergency requiring early diagnosis and urgent management, which can be challenging due to its diverse clinical presentations. While common presentations include fever, sweating, palpitations, tremors and confusion, presence of jaundice is rare.

**Case presentation:**

We report a 35-year-old male who presented with jaundice due to cholestasis along with other features of thyrotoxic crisis due to Graves’ disease. He had a good clinical recovery with resolution of cholestasis following treatment for thyrotoxic crisis.

**Conclusion:**

Jaundice can be a rare manifestation of thyrotoxic crisis, and should be considered in the differential diagnosis when other clinical features of thyrotoxic crisis are present. However secondary causes of jaundice should be looked into and excluded.

**Electronic supplementary material:**

The online version of this article (doi:10.1186/s13104-016-2126-z) contains supplementary material, which is available to authorized users.

## Background

Thyrotoxic crisis is an acute life threatening hyper-metabolic state precipitated by excessive release of thyroid hormones. Fever, tachycardia and hypertension with neurological and gastrointestinal symptoms are the commonly encountered clinical features. Thyrotoxic crisis warrants urgent diagnosis and commencement of treatment in order to reduce associated mortality.

Although hepatic dysfunction can be associated with thyrotoxic crisis, it is important to ascertain the etiology of jaundice and liver derangement as drug induced cholestasis, autoimmune liver disease and sepsis related hepatic dysfunction warrant specific management.

## Case presentation

A 35-year-old Asian male presented with fever, jaundice, loose stools, palpitations, bilateral ankle swelling, shortness of breath, agitation and tremulousness following a brief illness with cough and cold. He was diagnosed to have thyrotoxicosis due to Graves’ disease 2 years back and had defaulted treatment with carbimazole and propranolol after 6 months. He did not give a history of previous hepatitis infections or blood transfusions. He had no exposure history for leptospirosis or other etiological agents of acute hepatitis such as intravenous drug abuse, risky sexual encounters or consumption of street food. He denied any hepatotoxic drug treatment, and had no history of joint pains or facial rashes. He had no past history of other chronic diseases. He consumed alcohol occasionally and the last alcohol consumption was about 2 months prior to his current presentation.

On admission, he was febrile (101.6 °F) and had dyspnoea, deep icterus, tremulousness, sweaty skin and bilateral pitting ankle oedema. He had exophthalmos but no proptosis, lid lag or external opthalmoplegia. The thyroid gland was diffusely enlarged and there was no lymphadenopathy. He was conscious and rational with no focal neurological deficits. His Jugular venous pressure was elevated 8 cm above the sternal angle and the pulse rate was 120/min and irregularly irregular. The blood pressure was 150/70 mmHg. Examination of the lungs revealed end inspiratory bi-basal fine crepitations. The examination of abdomen was unremarkable except for mild tenderness over right hypochondrium.

His laboratory investigation results were as follows; white cell count was 2.9 × 10^3^/mL, platelets 59 × 10^3^/mL, haemoglobin 11.1 g/dL, haematocrit 32.5 % suggesting bi-cytopenia and blood picture had no evidence of microangiopathic haemolytic anaemia. Urine full report was normal except for a trace of albumin. Serum sodium 137 mmol/L, potassium 3.9 mmol/L and creatinine 64 µmol/L (60–115). The liver functions; aspartate aminotransferase (AST) 71 U/L (0–40), alanine aminotransferase (ALT) 35 U/L (0–40), alkaline phospatase (ALP) 259 U/L (34–104), total bilirubin 72.9 µmol/L (1–23.9), direct bilirubin 39.2 µmol/L, γglutamyltransferase 149 U/L (7–50) suggesting cholestasis. His blood culture was sterile. Serum calcium 2.04 mmol/L (2.10–2.54) and magnesium 0.8 mmol/L (0.7–0.9). C-reactive protein 10.64 mg/L (0.00–5.00) and ESR 42 mm/1st h. His thyroid stimulating hormone (TSH) 0.01 mIU/L (0.35–4.94), free T4 5.14 ng/dL (0.70–1.48). Ultrasound scan of neck revealed defuse enlargement of thyroid gland with no nodules or enlarged cervical lymph nodes. TSH receptor antibodies were positive with a titre of 6.08 U/L (positive >1.75), suggesting thyrotoxicosis secondary to Graves’ diseases. He was negative for hepatitis A IgM, hepatitis B surface antigen (HBsAg), cytomegalo virus (CMV) IgM, Epstein barr (EBV) IgM, leptospirosis antibodies and anti-nuclear antibodies. He was not checked for Hepatitis E as it is extremely rare in Sri Lanka. The ECG showed atrial fibrillation with rapid ventricular response, chest radiography showed cardiomegaly with upper lobe diversion and 2D echocardiography revealed global cardiac dysfunction with ejection fraction of 50 %, suggesting possible thyrotoxic cardiomyopathy. Serial ECGs did not show evidence of dynamic ischemic changes and cardiac biomarkers were negative for an acute coronary event. He was treated as thyrotoxic crisis with atrial fibrillation leading to heart failure precipitated by lower respiratory tract infection in addition to deranged liver functions with no acute liver failure. Therefore the medical management included oral high dose carbimazole (45 mg/day), intravenous (iv) hydrocortisone 100 mg 8 hourly, iv cefuroxime 750 mg 8 hourly, and oral clarithromycin 500 mg twice daily and oral bisoprolol 5 mg twice daily. His clinical condition and liver biochemistry improved with total bilirubin coming down to 46.6 µmol/L, AST 27 U/L, ALT 23 U/L after 1 week of treatment for thyrotoxicosis (Table 1). He was lost to follow up after 4 weeks and it was very sad to hear that he had succumbed to a fatal road traffic accident while attending to personal activities.

**Table 1:** Serial biochemical investigations

Investigation	Admission	Day 3	Day 7	Day 14
AST (u/L)	71	45	27	24
ALT (u/L)	35	33	23	24
Total bilirubin (umol/L)	72.9	64.3	46.6	25
TSH (mIU/L) (0.35–4.94 mIU/L)	0.01			
Free T4 (0.70–1.48 ng/dL)	5.14			

## Discussion

This patient had evidence of cholestatic jaundice together with thyrotoxic crisis. Occurrence of jaundice is rare with thyrotoxic crisis and can be due to various related or unrelated conditions. Although this can be due to thyrotoxic crisis itself, drugs that are used in the treatment of hyperthyroidism, other associated autoimmune disorders such as auto immune hepatitis (AH) or primary biliary cirrhosis (PBC), co-existing disorders such as Gilbert’s syndrome, thyrotoxic crisis associated cardiac failure causing hepatic congestion or atrial fibrillation resulting in hepatic artery clot-embolization may account for jaundice. However causes that are unrelated to hyperthyroidism such as viral hepatitis, alcohol abuse, sepsis, cholangitis and medications such as acetaminophen, isoniazid and rifampicin may complicate the picture with jaundice.

Although this patient had an acute respiratory tract infection prior to the onset of the illness it is unlikely that it would have resulted in a cholestasis. Furthermore he had not received any antibiotic such as co-amoxiclav that would cause cholestasis. He was also negative for common hepatitis viruses in Sri Lanka such as hepatitis A, B and other hepatotropic viruses such as EBV and CMV. Although leptospirosis gives a cholestatic pattern this possibility was excluded by serology.

Graves’ disease can be associated with autoimmune hepatitis (AIH) and primary biliary cirrhosis (PBC). While AIH is commoner in young patients, mainly females, PBC is commoner in middle aged females [1, 2] and they generally present with elevation of hepatic transaminases, alkaline phosphatase and gamma glutamyltransferase enzymes. Confirmation of AIH and PBC require presence of anti-nuclear antibodies and other liver specific antibodies like anti-smooth muscle antibodies and anti-mitochondrial antibodies. However anti-nuclear antibodies were negative in this patient making AIH and PBC unlikely. Furthermore his rapid response to short course of treatment with low dose steroids make an autoimmune aetiology for cholestasis unlikely.

In Gilbert syndrome, there is mild elevation of unconjugated bilirubin without hemolysis or transaminitis and is found in 2–13 % of the population [3]. Although they can become clinically icteric during stressful disorders, they will continue to have unconjugated hyper bilirubinaemia rather than rise in conjugated bilirubin. Therefore it is unlikely that this patient had co-existent Gilbert syndrome.

Furthermore, altered liver functions are known to occur in cardiac failure by two main mechanisms. They include liver congestion (congestive hepatomegaly) due to severe right heart failure or ischemic hepatitis [4]. Congestive hepatomegaly is often associated with moderately elevated transaminases; two to three times the upper limit and an increase in both the direct and indirect bilirubin [4]. Clinically these patients have features of severe right heart failure with elevated JVP, gross oedema and ascites [4]. However, in congestive hepatomegaly jaundice is a rare finding and the total bilirubin level is rarely greater than 50 µmol/L. This patient was icteric and had an AST level about two times normal, a normal ALT level and serum bilirubin level that is higher than what is expected in congestive hepatomegaly. Furthermore his Echocardiographic assessment did not reveal severe cardiac dysfunction or right heart failure. Therefore, it is unlikely that the hepatic involvement and icterus in this patient were due to congestion. On the other hand, the hallmark findings of ischemic hepatitis are severe jaundice, with a bilirubin level as high as 250 µmol/L, elevation of AST to more than ten times the upper reference range limit [4]. Therefore, in this patient the clinical picture and biochemical investigations were not suggestive of liver involvement secondary to cardiac dysfunction.

Primary hepatic dysfunction was described in patients with hyperthyroidism for the first time in 1874 [5]. Although there have been further descriptions of similar hepatic dysfunction in thyrotoxicosis the reasons behind such involvement are yet to be understood. There are several hypotheses proposed as the cause of cholestasis in thyrotoxicosis. Increased hepatic oxygen consumption without a parallel increase in hepatic blood flow in hypermetabolic state of hyperthyroidism may result in subsequent lowering of oxygen tension in the centrilobular zones. This may interfere with bile transport resulting in cholestasis [6]. However this hypothesis is yet to be proven.

Treatment with propylthiouracil has been reported to cause liver failure and deaths, especially in children [7]. However timely discontinuation of the drug leads to complete recovery in most cases [8]. With propylthiouracil treatment, a hepatic transaminitis with no elevation in bilirubin have been shown in about 30 % of patients after 2 months of therapy [9]. However in a majority of these patients normalization of transaminitis have been documented with further continuation of treatment for about 5 months [10]. On the other hand treatment with methimazole and carbimazole, has caused alteration in liver enzymes only in a few cases. Such alteration is thought to be due to a hypersensitivity reaction occurring in patients receiving normal doses of the drug and is usually associated with a cholestatic pattern in the liver function tests. It is known to occur within 2 weeks of initiation of therapy. Hepatic histology of such patients have shown cholestasis and non-specific infiltration of portal tracts with mono-nuclear cells and eosinophils [11].

This patient had a rapid improvement in clinical jaundice and cholestatic picture with specific management for thyrotoxic crisis. This suggest that thyrotoxic crisis itself as the most likely cause for his clinical picture.

## Conclusion

Thyrotoxic crisis, is a life threatening medical condition with a diverse clinical picture that require early diagnosis and treatment in order to reduce the associated mortality. Jaundice could rarely occur as part of clinical manifestation that improves with the specific management of thyrotoxic crisis alone. However careful evaluation in order to identify a secondary cause for jaundice may be warranted for the effective management.

We have adhered to the CARE guidelines for completeness and transparency of case reports (Additional file 1).

## Additional file

10.1186/s13104-016-2126-z CARE checklist.

## Abbreviations

AIH: autoimmune hepatitis
PBC: primary biliary cirrhosis
AST: aspartate aminotranferase
ALT: alanine aminotransferase
