# Supplementary material for: Thyrotoxic crisis presenting with jaundice
Source: BMC Res Notes. 2016 Jun 23;9:320. doi: 10.1186/s13104-016-2126-z (PMC4917949; doi:10.1186/s13104-016-2126-z)
Supplement: Supplementary file 1 — 10.1186/s13104-016-2126-z CARE checklist. [file 13104_2016_2126_MOESM1_ESM.docx]

**CARE Checklist – 2016: Information for writing a case report**

**Topic Item Checklist item description Line/Page**

**Title 1** The words “case report” should be in the title along with the area of focus 1/1

**Key Words 2** Four to seven key words—include “case report” as one of the key words 32/2

**Abstract 3a** Background: What does this case report add to the medical literature? 20/2

**3b** Case summary: chief complaint, diagnoses, interventions, and outcomes 24/2

**3c** Conclusion: What is the main “take-away” lesson from this case? 28/2

**Introduction 4** The current standard of care and contributions of this case—with references (1-2 paragraphs) 34/2

**Timeline 5** Information from this case report organized into a timeline (table or figure) _______

**Patient Information 6a** De-identified demographic and other patient or client specific information 45/3

**6b** Chief complaint—what prompted this visit? 45/3

**6c** Relevant history including past interventions and outcomes 45/3

**Physical Exam 7** Relevant physical examination findings 55/3

**Diagnostic 8a** Evaluations such assurveys, laboratory testing, imaging, etc. 63/4

**Assessment 8b** Diagnostic reasoning including other diagnoses considered and challenges 74/4

**8c** Consider tables or figures linking assessment, diagnosesand interventions 92/5

**8d** Prognostic characteristicswhere applicable 88/5

**Interventions 9a** Types such as life-style recommendations, treatments, medications, surgery 81/4

**9b** Intervention administration such as dosage, frequency and duration

**9c** Note changes in intervention with explanation 81/4

**9d** Other concurrent interventions ---------

**Follow-up and 10a** Clinicianassessment (and patient or client assessed outcomes when appropriate) 81/4

**Outcomes 10b** Important follow-up diagnostic evaluations 81/4

**10c** Assessment of intervention adherence and tolerability, including adverse events ----------

**Discussion 11a** Strengths and limitations in your approach to this case 94/5

**11b** Specifyhow this case report informs practice or Clinical Practice Guidelines (CPG) 139/7

**11c** How does this case report suggest a testable hypothesis? 139/7

**11d** Conclusions and rationale 164/10

**Patient Perspective 12** When appropriate include the assessmentof the patient or client on this episode of care

**Informed Consent 13** Informed consent from the person who is the subject of this case report is required by most journals 184/10

**Additional Information 14** Acknowledgement section; Competing Interests; IRB approval when required 199/10
